# Management of hepatocellular carcinoma, an important cause of death in Japanese autoimmune hepatitis patients

**DOI:** 10.1186/s12876-024-03204-z

**Published:** 2024-04-01

**Authors:** Tomoko Tadokoro, Takako Nomura, Koji Fujita, Takushi Manabe, Kei Takuma, Mai Nakahara, Kyoko Oura, Shima Mimura, Joji Tani, Asahiro Morishita, Hideki Kobara, Masafumi Ono, Tsutomu Masaki

**Affiliations:** 1 Department of Gastroenterology and Neurology, Kagawa University School of Medicine, 1750-1 Ikenobe, Miki-cho, Kita-gun, Kagawa, 761-0793 Japan; 2 Gastroenterology and Hepatology, HITO Medical Center, 788-1 Kamibun‐cho, Shikokutyuou, Ehime, 799‐0121 Japan; 3 Division of Innovative Medicine for Hepatobiliary and Pancreatology, Faculty of Medicine, Kagawa University School of Medicine, 1750-1 Ikenobe, Miki-cho, Kita-gun, Kagawa, 761-0793 Japan

**Keywords:** Autoimmune hepatitis, Hepatocellular carcinoma, Molecular-targeted drug, Immune checkpoint inhibitor, Carcinogenic factor

## Abstract

**Background:**

Hepatocellular carcinoma (HCC) in autoimmune hepatitis (AIH) was considered rare but is increasing with prolonged prognosis. Its impact on the overall prognosis of AIH is unknown, and treatment has not been established.

**Aim:**

To investigate the risk factors and prognosis of HCC in patients with AIH and identify appropriate management strategies.

**Methods:**

We studied patients with AIH including background liver disease, sex, age, complications, treatment, response to treatment, liver fibrosis, prognosis, and treatment.

**Results:**

In 131 patients, deaths due to liver failure were more common early after the onset of AIH; however, deaths due to HCC increased gradually. HCC was observed in 12 patients (median age, 70 years; male/female, 4/8; cirrhosis at onset, 11; median time to carcinogenesis, 7 years). Cirrhosis at diagnosis was identified as a risk factor for carcinogenesis in the multivariate analysis (odds ratio, 41.36; *p* < 0.0001) and cumulative cancer rates were high. Multidisciplinary therapy other than immune checkpoint inhibitors was administered as treatment for HCC. Two of the three patients who used molecular-targeted drugs discontinued the treatment because of adverse events.

**Conclusion:**

HCC is an important cause of death in patients with AIH. Currently available drug therapies are limited and early detection is desirable.

**Trial registration:**

This trial was retrospectively registered in the Ethics Committee of Kagawa University School of Medicine under the identifier 2019 − 238, registered on 4 Feb 2020.

## Introduction

Hepatocellular carcinoma (HCC) is the most frequent malignancy among primary hepatic cancers, an important cause of death, and is closely associated with chronic hepatitis. A variety of liver disorders can cause HCC, including viral hepatitis caused by hepatitis C virus (HCV) or hepatitis B virus, primary biliary cholangitis (PBC), autoimmune hepatitis (AIH), alcoholic liver disease, and nonalcoholic fatty liver disease/metabolic dysfunction-associated steatotic liver disease [1, 2]. Among these, HCC in autoimmune liver diseases, such as AIH, is conventionally considered rare [3]. The annual carcinogenic rate of AIH is estimated to be 0.19–0.53%, which is lower than that of viral hepatitis (0.37–4.81%) [1]. However, the carcinogenic rate has been increasing with prolonged prognosis, and has been reported in 5.1% of patients in Japan [4]. However, there are no established treatment recommendations for HCC in patients with AIH, and prognostic studies are scarce.

Immune checkpoint inhibitors, which are currently the central role in the treatment of HCC, are not recommended for patients with concomitant autoimmune diseases [5] and have not been studied in autoimmune liver diseases, limited to a few case reports on PBC [6] and no case reports on AIH.

This study retrospectively examined the carcinogenic factors, treatment, and prognosis in patients with AIH and examined the current appropriate management of HCC in AIH.

## Results

### Clinical characteristics

**Fig. 1:**
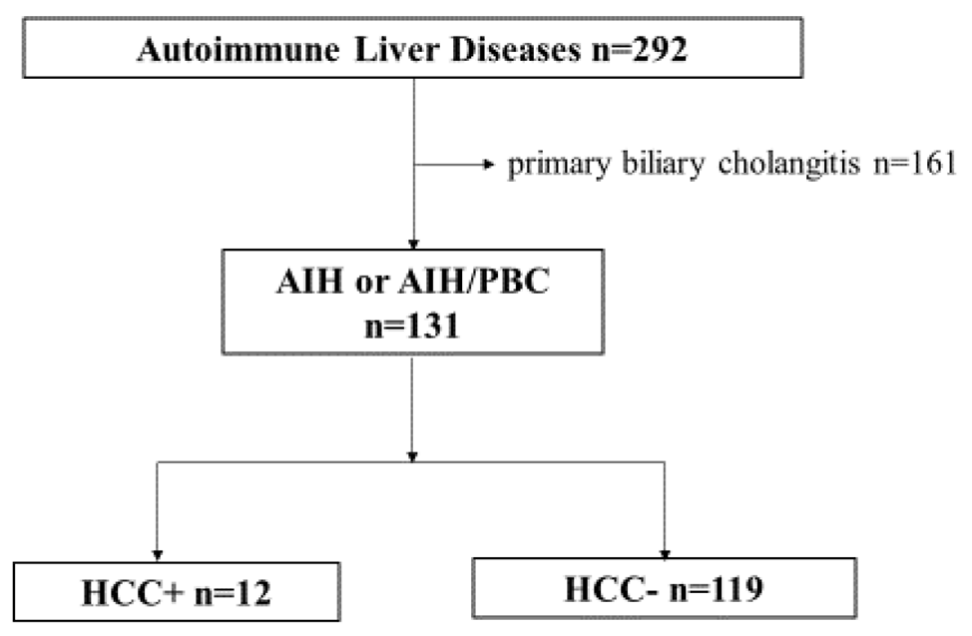
Flowchart of participants. AIH, autoimmune hepatitis; PBC, primary biliary cholangitis

In this study, 131 patients with AIH were enrolled and clinically analyzed. A flowchart of the patient selection process is illustrated in Fig. 1. The patients were classified according to whether they developed HCC during the observation period. The baseline patient characteristics are shown in Table 1. The following were the clinical characteristics of the study population: AIH/AIH-PBC: 87/44 cases, median age: 64 (52–71) years, male/female: 19/112 cases, median observation period: 1287 (256–3477) days, steroid use: 72 cases (55%), immunosuppressive drug use: 12 cases (9.2%), history of viral hepatitis: nine cases (6.9%) (active viral hepatitis or hepatitis on antiviral therapy: none), cirrhosis at diagnosis in 36 cases (27.5%), fatty liver at diagnosis in 37 cases (28.2%), diabetes mellitus at diagnosis in 19 cases (14.5%), body mass index > 25 at diagnosis in 38 cases (29.0%), history of blood transfusion in six cases (4.6%), history of alcohol consumption in 16 cases (12.2%), complicated gastroesophageal varices in 14 cases (10.7%), and HCC carcinogenesis in 12 cases (9.2%). There were 34 patients (26.0%) whose alanine transaminase (ALT) levels did not normalize after 1-year (ALT, 30 IU/L<) and 25 patients (19.1%) whose immunoglobulin G (IgG) levels did not normalize after 1 year (IgG, 1700 mg/dL<). Further, 27 cases (20.6%) had a high FIB-4 index (2.67≤) 1 year after diagnosis.

**Table 1:** The baseline patient characteristics and risk factors for hepatocellular carcinoma

Characteristic	HCC+ (*n* = 12)	HCC- (*n* = 119)	Univariate analysis			Multivariate analysis		
			odds ratio	95%CI	p	odds ratio	95%CI	p
autoimmune hepatitis	10	77	2.73	0.68–18.28	0.17			
elderly (≥ 65)	7	55	1.63	0.49–5.78	0.42			
liver cirrhosis at diagnosis	11	25	41.36	7.52-774.05	< 0.05	39.93	5.72-845.26	< 0.0001
male	4	15	3.47	0.93–12.93	0.08			
history of hepatitis virus	0	9	-	-	0.18			
duration of autoimmune hepatitis (≥ 10years)	3	29	1.03	0.26–4.08	0.96			
steroid use	8	64	1.72	0.49–6.18	0.39			
fatty liver	4	33	1.30	0.37–4.62	0.69			
alcohol	3	13	2.72	0.65–11.33	0.20			
diabetes	5	14	5.36	1.50-19.19	< 0.05	4.58	0.60-42.77	0.14
history of blood transfusions	1	5	2.07	0.22–19.36	0.55			
obesity	5	33	1.86	0.55–6.28	0.33			
Fib-4 index (≥ 2.67)	10	74	3.04	0.64–14.51	0.12			
ALT one year after diagnosis (≥ 31 IU/L)	7	27	5.44	1.40-26.75	< 0.05	3.12	0.56–21.46	0.21
IgG one year after diagnosis (≥ 1701 mg/dL)	4	21	2.33	0.64–8.47	0.21			
Fib-4 index one year after diagnosis(2.67≦)	7	20	8.17	1.93–34.50	< 0.05	2.13	0.36–13.79	0.40

Abbreviations: HCC, hepatocellular carcinoma; CI, Confidence Intervals; ALT, alanine transaminase; IgG, immunoglobulin G

### Overall survival and cause of death in patients with AIH

**Fig. 2:**
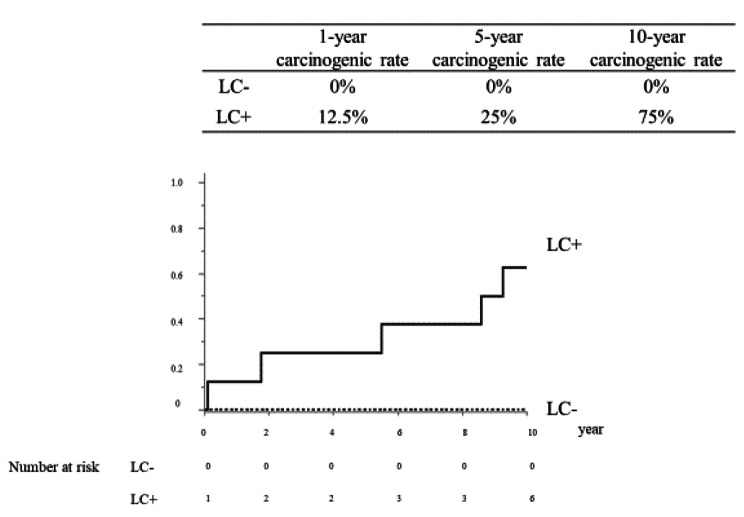
Carcinogenic rate of autoimmune hepatitis patients with or without liver cirrhosis. In the group with liver cirrhosis (LC), the 1-year, 5-year, and 10-year carcinogenic rates were 12.5% and 25% and 75% respectively. In the group without LC, one case of HCC was observed after more than 10 years. Three patients with hepatocellular carcinoma at the onset of AIH were excluded. LC, liver cirrhosis

The 1-year, 5-year, and 10-year survival rates of patients with AIH were 96.6%, 91.6%, and 85.6%, respectively. The median survival time (MST) was not achieved. In the group with liver cirrhosis, the 1-year, 5-year, and 10-year carcinogenic rates were 12.5%, 25.0%, and 75.0%, respectively. In the group without cirrhosis, one case of HCC was observed after 10 years (Fig. 2).

**Fig. 3:**
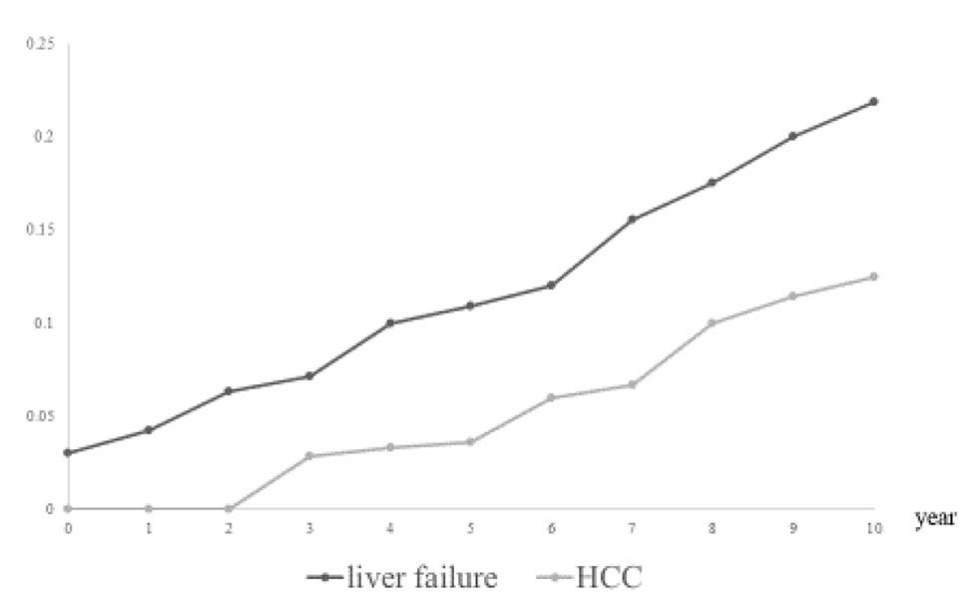
Cause of death by period. A review of the causes of death for the 14 patients who were confirmed dead during the observation period. The most common cause of death in patients with autoimmune hepatitis is liver failure in the early stages of the disease; however, deaths due to hepatocellular carcinoma have gradually increased. HCC, hepatocellular carcinoma

**Fig. 4:**
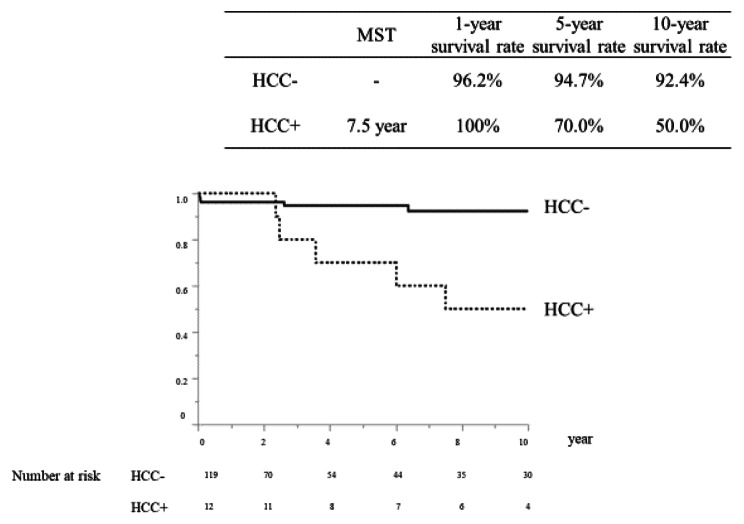
Overall survival rate of autoimmune hepatitis patients with or without hepatocellular carcinoma. In the group without HCC, the median survival time (MST) was not achieved and the 1-year and 5-year survival rates were 96.2% and 94.7%, respectively. In the group with HCC, the MST was 7.5 years, and the 1-year and 5-year survival rates were 100% and 70.0%, respectively. HCC, hepatocellular carcinoma

During the observation period, 21 patients with AIH died: nine from liver failure and five from HCC. Patients with liver failure could not undergo liver transplantation due to old age or lack of donors. Deaths due to liver failure were more common in the first year after AIH onset, but deaths from HCC later increased and tended to exceed those from liver failure (Fig. 3). Patients with HCC have significantly lower survival rates than those without HCC. In the group without HCC, the MST was not achieved and the 1-year and 5-year survival rates were 96.2% and 94.7%, respectively. In the group with HCC, the MST was 7.5 years, and at the 1-year and 5-year survival rates were 100% and 70.0%, respectively (Fig. 4).

### Characteristics and risk factors of HCC

HCC cases consisted of AIH/AIH-PBC: 10/2, median age: 70 years, male/female: 4/8, cirrhosis at onset: 11, median time to carcinogenesis: 7 (0–11) years, and four cases of HCC were found at the same time AIH was detected. There were no previous cases of viral hepatitis, fatty liver complication: four cases, drinking history: three cases, diabetes complication: five cases, blood transfusion history: one case, and steroids were used as treatment in eight cases and immunosuppressive drugs in one case according to AASLD guidelines [7]. Their biochemical remissions reached 30%.

In the univariate analysis, cirrhosis at diagnosis, concomitant diabetes mellitus, and high ALT levels and FIB-4 index at 1 year were identified as risk factors for HCC, whereas in the multivariate analysis, cirrhosis at diagnosis was identified as a risk factor for HCC (Table 1). Gastroesophageal varices as an objective indicator of cirrhosis were also extracted as a risk factor for HCC in a multivariate analysis (*p* = 0.0001, odds ratio 18.33, 95%CI:4.14-102.26). Steroid or immunosuppressive drug use as a treatment strategy for AIH was not identified as a risk factor. In the old cases, there were no cases with diabetes mellitus or male patients, while in the recent cases, 55% had diabetes mellitus and 44% were male patients.

### Treatment and prognosis of HCC

HCC progressed to stage I, II, III, and IV in 3, 5, 4, and 0 cases, respectively. Initial treatment included surgery in two cases, thermal ablation in six cases, and transarterial chemoembolization in four cases. Three months after the initial HCC treatment, mRECIST was used to determine the best treatment effect based on contrast-enhanced CT findings. The overall response rate (CR + PR) was 58.3%. The disease control rate (CR + PR + SD) was 66.7%. The two patients who underwent surgery had moderately differentiated HCC. Of the 12 HCC patients, 8 had recurrence within the observation period. Molecular-targeted drugs were used in three patients after second-line treatment; however, sorafenib was administered in two patients, one discontinued due to hypothyroidism and the other due to fatigue. One patient treated with lenvatinib had only grade 1 hand-foot syndrome, and the treatment effect was a PR. No cases of immune checkpoint inhibitor use were observed. No AIH relapses occurred during treatment (Table 2).

In the group with HCC, the 1-year, 5-year, and 10-year survival rates were 100.0%, 53.6%, and 17.9% respectively.

**Table 2:** Characteristics and treatment of hepatocellular carcinoma with autoimmune hepatitis

primary disease	sex	age at HCC	duration to carcinogenesis (year)	HCC stage	initial treatment	initial treatment effect	molecular-targeted drugs
AIH	male	73	10	3	TACE	CR	intolerance
AIH-PBC	male	77	0	3	TACE	SD	-
AIH	male	71	14	2	TACE	CR	PR
AIH	female	79	20	1	thermal ablation	PD	-
AIH	female	76	0	3	surgery	PD	intolerance
AIH-PBC	female	71	8	1	thermal ablation	CR	-
AIH	female	54	14	2	thermal ablation	CR	-
AIH	male	70	1	1	TACE	PD	-
AIH	female	79	5	2	thermal ablation	CR	-
AIH	female	73	0	3	TACE	-	-
AIH	female	71	0	2	surgery	CR	-
AIH	female	80	9	2	thermal ablation	CR	-

Abbreviations; HCC, hepatocellular carcinoma; CR, complete response; PR, partial response; SD, stable disease; PD, progressive disease.

AIH, autoimmune hepatitis; AIH-PBC, autoimmune hepatitis-primary biliary cholangitis overlap syndrome: TACE, transarterial chemoembolization.

## Methods

### Patients

A retrospective observational study was conducted in 131 patients with a history of AIH or AIH-PBC overlap syndrome (AIH-PBC) between January 2001 and December 2022 at the Department of Gastroenterology, Kagawa University. The patient background was examined with regard to sex, height, weight, age at diagnosis, comorbidities (especially autoimmune diseases), presence of viral hepatitis, presence of fatty liver (diagnosed on imaging or histologically), history of alcohol consumption, diabetes, blood transfusions, treatment (steroids, immunosuppressive drugs, among others), carcinogenesis, prognosis, cause of death, risk factors for carcinogenesis, cancer treatment, and course after carcinogenesis. Patient backgrounds of HCC cases were compared between old cases (2001–2011) and recent cases (2012–2022).

### Diagnosis of disease

AIH was diagnosed using the revised scoring system by International Autoimmune Hepatitis Group Report [8]. In addition, it was confirmed that the patients also met the AIH diagnostic criteria in simplified diagnostic criteria for AIH [9]. The Paris criteria were used to diagnose AIH-PBC [10]. Liver cirrhosis was comprehensively diagnosed using criteria such as morphological changes in the liver by computed tomography (CT) or abdominal ultrasonography, reduced platelet count (less than 140 × 10^9^/L), presence of gastroesophageal varices, and significant fibrosis by biopsy of the liver. Hepatitis B patients were identified as those positive for hepatitis B surface antigen or with a history of hepatitis B. Hepatitis C patients were identified as those positive for HCV antibodies or HCV RNA or with a history of hepatitis C. Non-hepatitis B and non-hepatitis C patients were identified as those who did not fit into any of the above groups. The presence of fatty liver was determined using CT imaging, abdominal ultrasonography, or histology. Drinking alcohol was defined as having a daily net alcohol intake of 30 g or more for men and 20 g or more for women. Diabetes mellitus was defined as a history of hypoglycemia or dietary guidance. A body mass index of 25 or higher was defined as obese. The elderly patients were defined as ≥ 65 years of age. The fibrosis index based on four factors (FIB-4 index) was used as a measure of fibrosis [11]. The definition of HCC was confirmed using supplemental tumor markers such as α-fetoprotein and des-γ-carboxyprothrombin, abdominal ultrasonography, contrast-enhanced CT and magnetic resonance imaging, and if typical HCC findings were not obtained by these tests, a needle biopsy was performed to confirm the diagnosis. The clinical stage of HCC was assessed based on the size, number, vascular invasion, lymph node metastasis, and metastasis at distant sites, using the tumor-lymph node-metastasis classification based on the criteria of the Liver Cancer Study Group of Japan [12]. Treatment response was classified as complete response (CR), partial response (PR), stable disease (SD), or progressive disease according to the modified Response Evaluation Criteria in Solid Tumors (mRECIST) based on contrast CT findings [13]. Adverse events were assessed drug therapy according to the Common Terminology Criteria for Adverse Events version 5.0.

### Statistical analysis

Continuous variants are indicated by median values, with ranges shown in parentheses. Categorical variables are expressed as numbers and percentages. Comparisons among the two groups were performed using the t-test, chi-square test, and Fisher’s exact probability test. Representative results are expressed as medians (interquartile range). Survival analysis was conducted with the Kaplan–Meier method or Cox proportional hazards model. Statistical significance assumed to be *p* < 0.05. All statistical analyses were conducted using JMP Pro 17.0(SAS Institute, Cary, NC, USA). Factors reported to be associated with HCC include cirrhosis [14–16], older age [17], male age, characteristics of portal hypertension, history of blood transfusion [14], repeated recurrence of AIH [18, 19] and sustained elevation of serum ALT level during follow-up [20]. With reference to these reports, age, gender, history of blood transfusion, presence of cirrhosis, varices, and liver function (ALT, IgG, Fib-4index) during the course of the study were selected as independent variables. In addition, hepatitis virus, diabetes mellitus, obesity, and alcohol consumption, which are generally considered risk factors for HCC, were also selected as independent variables.

## Discussion

In this study we investigated the risk factors and prognosis of HCC in patients with AIH and identified appropriate management strategies. Japanese patients with AIH have good outcomes, and the overall survival of all patients in this study was favorable, consistent with previous reports [14]. However, patients with HCC in the course of the disease had significantly lower survival rates than those without HCC. The 1- and 5-year survival rates for HCC in general were 88.9% and 56.2% [21] and are similar to those observed in this study. Although reports on the prognosis of patients with AIH indicate that death within 1 year of onset and liver transplantation are common [22] and liver failure is an important cause of death, there are few reports on the importance of HCC as the cause of death when considering long-term prognosis. This study found that management of HCC is necessary for patients with AIH who survive the acute phase of the disease.

In a previous report, the carcinogenic rate of viral hepatitis was high due to differences in the immunological profile of regulatory T cells and other factors [23], and carcinogenesis in AIH was considered rare [1]. However, the present report suggests that the rate of HCC carcinogenesis in AIH patients may increase over time. The most important HCC risk factor for AIH in this study was cirrhosis at diagnosis, which is similar to the findings of previous reports [1, 4]. In this study, the diagnosis of cirrhosis was made with reference to imaging studies, histological image of the liver, and blood tests. Analysis of gastroesophageal varices as an objective indicator of cirrhosis was also consistent as a risk factor for HCC. On the other hand, the results for indices such as platelets and Fib-4 index based on blood data showed little association with HCC, suggesting the importance of imaging tests, since AIH is often accompanied by severe liver dysfunction at the onset, making it difficult to predict HCC based on blood data at the onset. Although the multivariate analysis did not reveal significant differences, patients with diabetes at diagnosis and no ALT normalization after 1 year may also require attention for HCC. They were receiving standard treatment, but their ALT levels had not normalized sufficiently. Persistent chronic inflammation may have influenced the development of HCC [23]. Although not statistically significant due to the small number of cases, the carcinogenic rate in males is as high as 21%, suggesting the need for caution in HCC as in the previous report [19]. In this study, there were no cases with diabetes mellitus or male patients in the old cases, while 55% had diabetes mellitus and 44% were male patients in the recent cases. Because of the small number of cases, these differences were not statistically significant, but there may be an increase in carcinogenesis from men and diabetics.

In previous reports, the risk factors for carcinogenesis of AIH were two or more recurrences [18], male sex, portal hypertension, history of blood transfusion, immunosuppressive therapy for more than 3 years, therapeutic failure, liver cirrhosis for more than 10 years [19], advanced age, and elevated ALT levels during treatment [14, 17, 20]; however, there is still no consensus. In a recent multicenter study, the risk factors for HCC in AIH were obesity at baseline, cirrhosis, and AIH-primary sclerosing cholangitis overlap syndrome [15]. Patients with AIH with cirrhosis, which is an overwhelming risk factor based on previous reports and in this study, should be considered a high-risk group. Therefore, abdominal ultrasonography and serum alpha-fetoprotein measurements should be performed every 6 months [5, 7]. AIH management requires the appropriate use of first-line steroids to normalize ALT and IgG [24]. However, there was some debate regarding steroids as a risk factor for HCC [16], according to some reports steroids are unlikely to induce HCC [4, 14], and the association between steroid administration and HCC was also scant in the present study. In Japan, steroid-resistant AIH is found in approximately 10% of the cases, where azathioprine is used [4]. There have been reports of HCC in cases of long-term azathioprine use in Crohn’s disease and other inflammatory bowel diseases [25]. Azathioprine and AIH carcinogenesis are also of interest; however, in this and previous studies, the association between AIH and carcinogenesis was poor [4]. In HCC with AIH, because the state of cirrhosis is considered to be closely related to HCC, it is desirable to stop the progression to cirrhosis with the aim of ALT normalization without hesitation regarding the use of steroids and immunosuppressive drugs.

The most important problem with HCC in AIH cases is the lack of therapeutic drug options. Immune checkpoint inhibitors, which are the first-line agents for unresectable HCC, are not recommended for AIH. Clinical trials assessing immune checkpoint inhibitor-based therapies exclude AIH patients with HCC. Thus, clinical data on the safety and efficacy of immune checkpoint inhibitors are lacking [26]. Treatment with immune checkpoint inhibitors may be considered for patients with a history of autoimmune disease if the autoimmune disease is mild or well-controlled and relapse is not potentially life-threatening [26]. However, autoimmune-related adverse events or relapse of liver disorder after treatment with immune checkpoint inhibitors can lead to life-threatening complications, such as liver failure. Thus, treatment based on immune checkpoint inhibitors should be attentively considered after assessing the possible risks and benefits. Transcatheter arterial chemoembolization using cisplatin and other drugs, as well as molecularly-targeted drugs, is still effective in the era of immune checkpoint inhibitors [27]. Currently, we provide multidisciplinary treatment without the use of immune checkpoint inhibitors. In contrast, two of the three patients who used molecular-targeted drugs in this study discontinued treatment because of adverse events. There are no reports on the use of molecular-targeted agents in patients with AIH; therefore, further investigation of the safety of these agents is needed.

This study has its limitation as it was a retrospective study with a limited number of participants. In addition, AIH/PBC patients may have a different molecular biological background than pure AIH, and therefore should be studied in pure AIH patients. Nevertheless, there have been few reports on clinical investigations of HCC in AIH, and we believe that this study is important because HCC may increase as the prognosis of AIH increases in the future. We believe that this study is useful in AIH practice, especially since there are few reports indicating that HCC is an important cause of death in AIH, and few reports mention the treatment of HCC, especially with molecularly-targeted drugs.

In conclusion, HCC has become an increasingly important cause of death in patients with AIH. Currently, there is no established drug therapy, and surveillance of HCC is important, especially in high-risk patients such as those with cirrhosis.

## Data Availability

The data that support the findings of this study are not openly available due to reasons of sensitivity and are available from the corresponding author upon reasonable request. Data are located in controlled access data storage at Department of Gastroenterology and Neurology, Kagawa University.
